# The New Harvard Veterinary School

**Published:** 1883-10

**Authors:** 


The New Harvard Veterinary School.—A writer in the *Boston Medical* and *Surgical Journal*, has taken the trouble to "write up" the new Veterinary College connected with Harvard University. Everything is naturally viewed in a very rosy light, and we are led to suppose that the Boston school will at once take the lead of all veterinary colleges. Some exhibition of local pride is of course to be expected ; but it was hardly necessary in thus "touting" Harvard to speak slightingly and ignorantly of the New York institutions.

Those acquainted with the workings of Columbia Veterinary College, for example, know that it requires a longer term than other New York Veterinary Colleges, that evidence of preliminary education is demanded, and that no school in this country does or can give its students so thorough a drill in all the fundamental branches of comparative medicine. Boston may have a magnificent hospital (with accommodations for eleven horses), but the students of the Columbia College have access to six.

Harvard has no doubt a fine school, but it is young, and those connected with it will learn in time that building horse-hospitals and drawing up finely worded circulars, do not start a veterinary college.

For one thing it is not easy to get good teachers and New York has had to educate hers. Boston will be able to do so in time.

Columbia Veterinary College has been the centre for several years of sincere earnest work in comparative pathology and physiology, and it deserves some credit and recognition of the fact. This the hasty and exuberant correspondent of the *Boston Medical* and *Surgical Journal*, fails to give.

